# YTHDF2 promotes intrahepatic cholangiocarcinoma progression and desensitises cisplatin treatment by increasing CDKN1B mRNA degradation

**DOI:** 10.1002/ctm2.848

**Published:** 2022-06-13

**Authors:** Chen‐Song Huang, Ying‐Qin Zhu, Qiong‐Cong Xu, Siyun Chen, Yue Huang, Guangyin Zhao, Xuhao Ni, Bo Liu, Wei Zhao, Xiao‐Yu Yin

**Affiliations:** ^1^ Department of Pancreato‐Biliary Surgery The First Affiliated Hospital, Sun Yat‐sen University Guangzhou China; ^2^ Key Laboratory of Stem Cells and Tissue Engineering Ministry of Education Sun Yat‐sen University Guangzhou China; ^3^ Department of Biotherapy Sun Yat‐sen University Cancer Center Guangzhou China; ^4^ Department of Animal Experiment Center The First Affiliated Hospital, Sun Yat‐sen University Guangzhou China

**Keywords:** cisplatin‐resistance, intrahepatic cholangiocarcinoma, tumourigenensis, YTHDF2

## Abstract

**Background:**

Intrahepatic cholangiocarcinoma (ICC) is an aggressive cancer with exceedingly poor prognosis, and chemoresistance is a huge challenge for treatment. N6‐methyladenosine (m^6^A) modification plays an important role in the progression and chemoresistance of cancers. We aimed to investigate the oncogenic function and therapeutic significance of the m^6^A binding protein, YTH domain family 2 (YTHDF2), in ICC progression and cisplatin‐based chemotherapy.

**Methods:**

Several independent data sets were used to assess the expression of YTHDF2 in ICC, particularly in chemoresistant ICC. Knockdown and overexpression were used to evaluate the effects of YTHDF2 on tumourigenesis and cisplatin response in ICC. Multi‐omics sequencing was performed to identify target genes. RIP, dual luciferase reporter, RNA stability experiment and loss‐of‐function assays were conducted to study the mechanisms underlying the oncogenic function of YTHDF2. Furthermore, patient‐derived xenograft (PDX) model was established to determine the effect of combination treatment of *YTHDF2* siRNA and cisplatin in ICC.

**Results:**

Our study showed that YTHDF2 was upregulated in ICC tissues, particularly in chemoresistant ICC tissues, and correlated with poor prognosis. Furthermore, silencing YTHDF2 led to inhibited proliferation, promoted apoptosis and G0/G1 cell cycle arrest. Its downregulation also enhanced DNA damage and sensitised ICC cells to cisplatin. YTHDF2 overexpression exerted the opposite results. Integration analysis using RNA‐seq, MeRIP‐seq and anti‐YTHDF2 RIP‐seq elucidated the role of YTHDF2 in tumourigenesis and cisplatin‐desensitising function by promoting the degradation of cyclin‐dependent kinase inhibitor 1B (CDKN1B) mRNA in an m^6^A‐dependent manner. Downregulation of CDKN1B increased the YTHDF2 silencing‐induced influence on tumourigenesis and cisplatin response to ICC. In addition, the combination treatment of *YTHDF2* siRNA and cisplatin significantly enhanced the anti‐tumour effect of cisplatin in a chemoresistant ICC PDX model.

**Conclusions:**

YTHDF2 exhibits tumour oncogenic and cisplatin‐desensitising properties, which may offer insight into the development of novel combination therapeutic strategies for ICC.

## Graphical abstract

- YTHDF2 is upregulated in ICC tissues, particularly in chemoresistant ICC tissues, and correlated with poor prognosis.
- Overexpression of YTDHF2 promotes ICC cell proliferation, inhibits apoptosis and accelerates cell cycle.
- Overexpression of YTHDF2 desensitised ICC cells to cisplatin treatment
- YTHDF2 facilitates ICC progression and desensitised cisplatin treatment by downregulating CDKN1B expression in an m^6^a dependent manner.

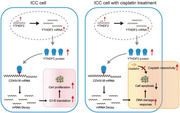

## 1 INTRODUCTION

Intrahepatic cholangiocarcinoma (ICC) ranks the second most common primary liver cancer after hepatocellular carcinoma, accounting for 10–15% of primary liver cancer.^1^, ^2^ The incidence rate and mortality rate of ICC have recently been rising globally,^3^ and surgical resection is currently the treatment of choice. However, few patients are eligible for surgery due to rapid progression and metastasis. Also, the 5‐year survival rate of surgically resected ICC patients is only 25–40% owing to the high recurrence rate.^4^ Cisplatin‐based combination therapy has been used as the first‐line treatment for patients with ICC presenting with locally advanced or distal metastasis.^5^, ^6^ However, insensitivity to drugs or the rapid development of chemoresistance could lead to poor results. Therefore, identifying new targets of carcinogenesis in ICC or elucidating the molecular mechanisms underlying cisplatin insensitivity and resistance will improve the life expectancy of patients with ICC.

Epigenetic alterations play vital roles in tumourigenesis, chemoinsensitivity and anti‐tumour immunity.^7^, ^8^, ^9^ N6‐methyladenosine (m^6^A) modification is the most abundant mRNA modification in eukaryotes, occurring in ∼30% of transcripts at the whole genome level.^10^ m^6^A modification is dynamic and reversible and regulated by methylases, demethylases, and specific ‘reader’ proteins.^11^ The dysregulation of m^6^A modification affects various aspects of RNA processing, including splicing, export, degradation and translation, which lead to a series of diseases, particularly cancers.^12^, ^13^

The functions of m^6^A methyltransferases (writers) and demethylases (erasers) have been widely defined in a variety of cancers. But the diverse outcome of post‐transcriptional regulation mostly depends on the m^6^A readers, which are widely studied in recent years. The currently confirmed readers include YTH domain proteins (YTHDF1, YTHDF2, YTHDF3, YTHDC1 and YTHDC2) and HNRNP family proteins.^12^ Among them, YTH domain family 2 (YTHDF2) is the most effective m^6^A reader, exhibiting a series of biological processes involved in cancer carcinogenesis.^14^ As a main cytoplasm m^6^A reader, the major function of YTHDF2 is mediating mRNA degradation by recognising m^6^A modification in the 3′‐UTR of mRNA and promoting the tumour progression in several solid tumours^15^, ^16^, ^17^, ^18^ and acute myeloid leukaemia.^19^ However, whether YTHDF2 is involved in tumourigenesis and cisplatin‐desensitising function in ICC remains unclear. We herein report that the m^6^A reader YTHDF2 is upregulated in patients with ICC, especially in chemoresistant ICC tissues, and correlated with poor prognosis. Overexpresssion of YTHDF2 promotes proliferation, inhibits apoptosis, decreases the arrest of the G0/G1 stage and desensitises ICC cells to cisplatin, which is dependent on the degradation of cyclin‐dependent kinase inhibitor 1B (*CDKN1B*) mRNA in an m^6^A‐dependent manner. Finally, the combination treatment of si*YTHDF2* and cisplatin enhanced the anti‐tumour effect of cisplatin in a chemoresistant patient‐derived xenograft (PDX) model of ICC.

## 2 MATERIALS AND METHODS

### 2.1 Patients’ specimens

A total of 118 paraffin‐embedded specimens, which were pathologically confirmed as ICC, were obtained from the pathology department of the First Affiliated Hospital of Sun Yat‐sen University (Guangzhou, China). Among them, 96 specimens from ICC patients undergoing operation and having complete clinical–pathological characteristics and follow‐up data were used for subsequent survival analysis. Another 22 specimens from unresectable ICC patients who received cisplatin + gemcitabine as chemotherapy in our hospital were recruited. The chemotherapy response was evaluated by computed tomography after two cycles of chemotherapy according to the Response Evaluation Criteria in Solid Tumors guidelines (RECIST, version1.1). Compete response (CR) and partial response (PR) were defined as chemosensitive patients. Stable disease (SD) and progression disease (PD) were defined as chemoresistant patients. Another 10 pairs of ICC tissues and adjacent liver tissues were collected for detecting the mRNA expression level of *YTHDF2*. The animal studies were conducted by the approval of the First Affiliated Hospital of Sun Yat‐sen University Ethics Committee ([2020] No.338).

### 2.2 Public data sets analysis

We analysed the *YTHDF2* mRNA level in ICC using The Cancer Genome Atlas (TCGA) database including 30 ICC and 8 adjacent non‐cancerous tissues samples. The gene expression profiles of GSE107943 and GSE107101 from the Gene Expression Omnibus (GEO) database were also surveyed.

### 2.3 Cell culture

The ICC cell lines (HuCC‐T1, RBE and HCCC‐9810) and normal intrahepatic bile duct cell line (HIBEC) were purchased from Cellcook Co., Ltd. (Guangzhou, China). Cells were cultured in RPMI 1640 medium (Gibco, USA), containing 10% of FBS (Gibco, USA) at 37℃ in a humidity of 5% CO_2_.

### 2.4 Reagents and antibodies

The small interfering RNA (siRNA) of human *YTHDF2*, *CDKN1B* and non‐targeting control were purchased from RiboBio Co., Ltd. (Guangzhou, China). The short hairpin RNA (shRNA) targeting *YTHDF2* and primers for qRT‐PCR analysis were synthesised from Sangon Biotech Co., Ltd (Shanghai, China). The oligonucleotides sequences of the siRNAs and shRNAs were listed in Supplementary Table S1. The transfection of siRNA, shRNA and overexpression plasmids were performed as described previously.^20^ The sequences of the primers used for qRT‐PCR were also listed in Supplementary Table S1. Antibody against YTHDF2 (#24744‐1‐AP) was purchased from Proteintech. Antibody against Ki67 (#ab156956) was purchased from Abcam. The anti‐H2A.X phosphorylated antibodies (#613415) was purchased from Biolegend. Antibody against m^6^A (#202003) was from Synaptic Systems. Antibodies against CDK2 (#2546S), CDK4 (#12790S), cyclin D3 (#2936), cyclin E1 (#20808), p27 (#3686S), β‐actin (#4970), Caspase‐3 (#14220S), Cleaved Caspase‐3 (#9664S), Caspase‐9 (#9508S) and Cleaved Caspase‐9 (#52873S) were from Cell Signaling Technology. The Actinomycin D (#A9415) was from Selleck Chemicals (Houston, TX, USA).

### 2.5 Western blotting

Western blotting was conducted as described previously.^20^ In brief, cells were lysed in SDS loading buffer for immunoblotting. Protein samples were separated by gel electrophoresis, followed by transferring onto a PVDF membranes (Milipore, USA). After blocked with 5% milk in TBST, the membrane was incubated with primary antibodies (1:1000) and subsequently probed by corresponding secondary antibody (1:2000). The signals were detected using ECL Western Blotting substrate (Proteintech, USA).

### 2.6 Dual luciferase reporter assay

The indicated ICC cells were transfected with CDKN1B wild‐type or mutant dual‐luciferase reporter plasmid. At 48 h post‐transfection, the luciferase intensity was detected according to manufacturer's instructions of the Dual‐luciferase Reporter Assay System (Promega, USA). The data were described in R‐Luc/F‐Luc value and the firefly luciferase served as the internal control.

### 2.7 RNA decay assays

After treated with 5 μg/ml actinomycin D for 0, 1, 2 and 3 h, the total RNA of ICC cells was collected and isolated using TRIzol reagent (Life Technologies, USA). The expression level was then detected by RT‐qPCR analysis.

### 2.8 Tumour xenograft models

For tumour xenograft models, 1 × 10^7^ HuCC‐T1 cells in knockdown group or control group were implanted into the right flank of 5‐week‐old female nude mice. The volumes of tumour were recorded every 4 days by calliper. The volumes were calculated as length × width^2^/2. The mice were executed at 32 days post‐implantation, and the tumours were photographed and weighed.

For patient‐derived xenograft (PDX) model (PDX0075), ICC tissues from a patient, who relapsed in 6 months after R0 resection and subsequent chemotherapy with cisplatin and gemcitabine, were diced into 3 mm^3^ pieces and transplanted subcutaneously into the right flank of 5‐week‐old female B‐NDG^®^ mice (Biocytogen, Beijing, China). When the xenografted tumours (Passage 1, P1) reached 1 cm^3^, we excised and cut the tumours into pieces, then further implanted them into B‐NDG^®^ mice for serial transplantation (Passage 2–3, P2–3). Then, the P3 tumours were implanted into 5‐week‐old female BALB/c nude mice. The volumes were measured by calliper and calculated as length × width^2^/2. When the tumour volumes reached 50 mm^3^, mice were randomly divided into four groups (siControl+vehicle, si*YTHDF2*+vehicle, siControl+cisplatin and si*YTHDF2*+cisplatin). Mice were intraperitoneally injected with vehicle (saline) or cisplatin (dissolved in saline, 4 mg/kg, once a week), and oligonucleotide (3 nmol per mouse, twice a week) was accordingly injected into the tumour. Tumour volume was recorded twice a week. At the end point of the experiment, tumours were excised and weighed. We also measured the concentrations of aspartate aminotransferase (AST), creatinine (CREA), alanine aminotransferase (ALT) and blood urea nitrogen (BUN) in the serum. Terminal deoxynucleotidyl transferase dUTP nick‐end labelling (TUNEL) assay were conducted to compare the apoptosis cells in different groups. The animal studies were conducted by the approval of the First Affiliated Hospital of Sun Yat‐sen University Ethics Committee ([2020] No. 383).

### 2.9 Immunohistochemistry (IHC)

Immunohistochemistry manipulation was conducted as previously described.^20^ Two experienced pathologists estimated the expression of indicated proteins and counted the IHC scores independently. We examined the protein expression according to staining intensity (negative, 0; weak, 1; intermediate, 2; strong, 3) and positive percentage (negative, 0; 1–30%, 1; 31–60%, 2; above 60%, 3). The IHC score was calculated by multiplying two scores above. The median is used to distinguish high and low expression.

### 2.10 Real‐time quantitative polymerase chain reaction (RT‐qPCR)

TRIzol reagent was used for total RNA isolation from indicated cells with different treatments. Reverse transcription and qPCR analysis were conducted using HiScript II Q RT SuperMix for qPCR (R223‐01, Vazyme, China) and SYBR qPCR Master Mix (Q711‐02, Vazyme, China), respectively. We then detected the relative expression levels using QuantStudio 6 Flex Real‐Time PCR Systems (Applied Biosystems, USA) and the data were analysed according to 2^−ΔΔCT^ methods.

### 2.11 RNA‐seq

The total RNA of transfected HuCC‐T1 cells was isolated using TRIzol solution. The following cDNA library construction and paired‐end sequencing were performed by Novogene (Beijing, China). The Agilent Bioanalyzer 2100 was used to analyse the integrity of the RNA. The paired‐end reads were calculated by the Illumina Novaseq 6000 and mapped to the human genome hg38 using hisat2. Then, the R package, DESeq2 (version: 1.34.0) was used to define differentially expressed genes with a cut‐off of *p* < .05. Gene Set Enrichment Analysis (GSEA) is supported by the Broad Institute website (http://www.broadinstitute.org/gsea/index.jsp) and performed using the Java GSEA implementation.

### 2.12 Methylated RNA immunoprecipitation sequencing (MeRIP‐seq) and MeRIP‐qPCR

Total RNA was extracted from ICC cells. After mRNA purification and fragmentation with dynabeads mRNA Purification Kit (61006, Invitrogen, USA) and RNA fragmentation reagent (AM8740, Invitrogen, USA), the mRNA was mixed with the anti‐m^6^A antibody for immunoprecipitated. Both input and immunoprecipitation RNA samples were prepared for sequencing procedure by Novogene (Beijing, China) or qPCR analysis.

### 2.13 RNA immunoprecipitation (RIP‐seq) and RIP‐qPCR

HuCC‐T1 and HCCC‐9810 cells were transfected with indicated lentivirus and lysed in RIP buffer. The lysate was incubated with the anti‐YTHDF2 antibody for immunoprecipitation. The RNA samples were then processed with proteinase K and TRIzol reagent. The purified samples were prepared for sequencing by Novogene (Illumina HiSeq 2500 platform) or qPCR analysis.

### 2.14 Statistical analysis

We employed SPSS version 22.0 and GraphPad Prism 9.0 software for statistical analysis. We used Kaplan–Meier method for calculating the disease free survival (DFS) and overall survival (OS). The differences were compared using log‐rank test. The statistical data in our study were described as mean ± standard deviation (SD) and compared by *t*‐test or Fisher's exact test, and *p* < .05 were defined as statistically significant.

## 3 RESULTS

### 3.1 YTHDF2 expression is upregulated in ICC tissues and is negatively correlated with the prognosis of ICC patients

**FIGURE 1:**
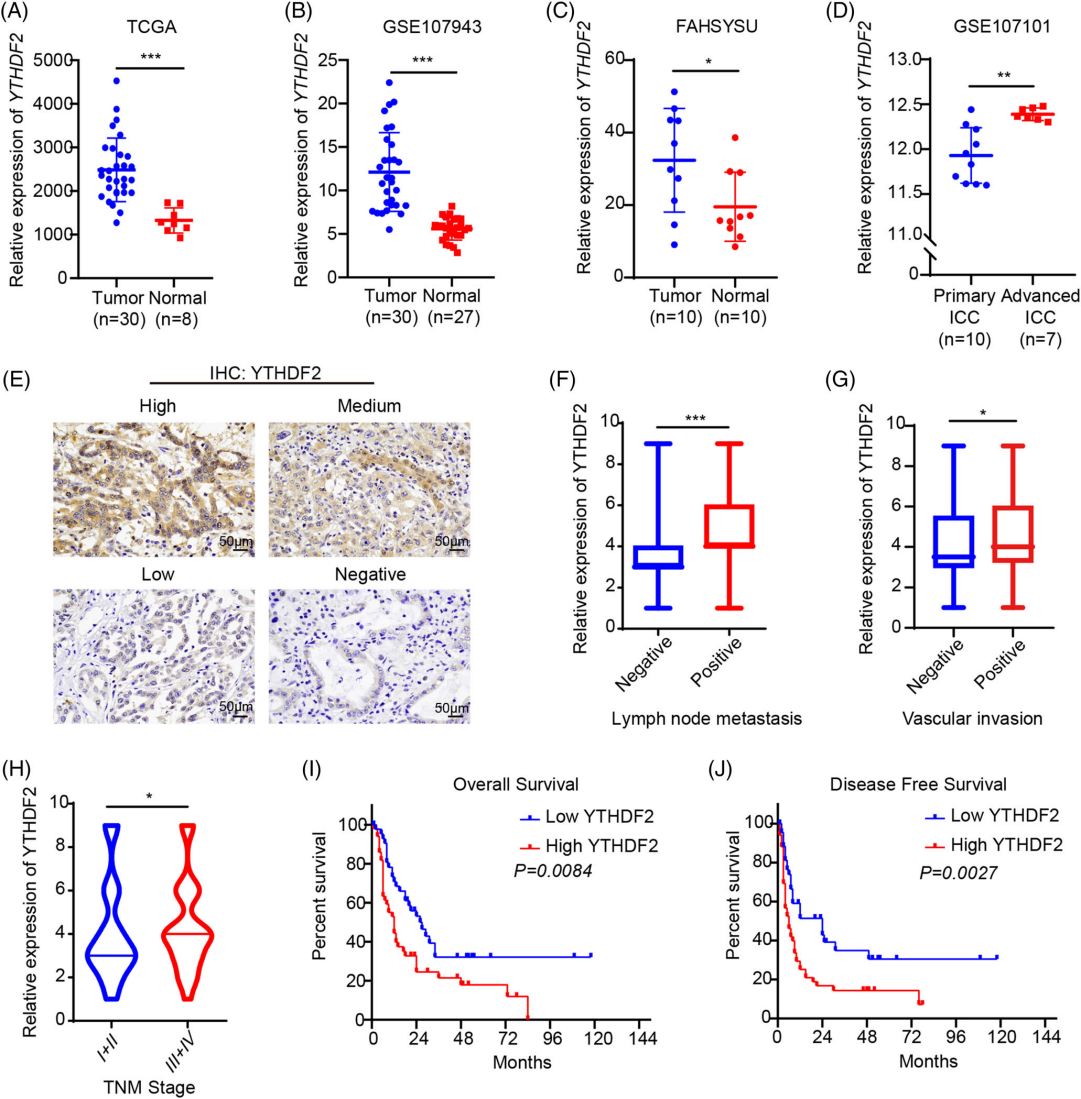
YTHDF2 expression is upregulated in ICC tissues and is correlated with poor prognosis of ICC patients. (A) YTHDF2 expression in ICC tumours (*n* = 30) and adjacent normal bile duct tissues (*n* = 8) from TCGA database. (B) YTHDF2 expression in ICC tumours (*n* = 30) and adjacent normal tissues (*n* = 27) from public data set GSE107943. (C) YTHDF2 expression in ICC tumours (*n* = 10) and adjacent normal bile duct tissues (*n* = 10) from our centre were measured by qRT‐PCR. (D) YTHDF2 expression of 10 primary ICC tumours and 7 advanced ICC tumours in public data set GSE107101. (E) Representative images of IHC staining for YTHDF2 in ICC tumours expressing low or high levels of YTHDF2. (F) Correlation analysis of YTHDF2 expression with lymph node metastasis (positive, *n* = 31) vs. negative, *n* = 65). (G) Correlation analysis of YTHDF2 expression with vascular invasion (positive, *n* = 17 vs. negative, *n* = 79). (H) Correlation analysis of YTHDF2 expression with TNM stage (stage I/II, *n* = 43 vs. III/IV, *n* = 53). (I) The overall survival curve in 96 ICC patients stratified by YTHDF2 IHC score was determined by Kaplan–Meier analysis (YTHDF2 low expression, *n* = 44 vs. YTHDF2 high expression, *n* = 52). The *p* value was calculated using the log‐rank test. (J) The disease‐free survival curve in 96 ICC patients stratified by YTHDF2 IHC score was determined by Kaplan–Meier analysis (YTHDF2 low expression, *n* = 44 vs. YTHDF2 high expression, *n* = 52). The *p* value was calculated using the log‐rank test. The data are presented as mean ± SD. A‐D are compared by *t*‐test. F–H are compared by Fisher's exact test. *p < .05; **p < .01; *** *p* < .001

First, we compared the expression of YTHDF2 between ICC tissues and adjacent non‐cancerous tissues in several public database. We found that YTHDF2 was upregulated in ICC tissues in both TCGA (Figure 1A) and GEO data set (GSE107943, Figure 1B), which was further validated in our local data (Figure 1C). Moreover, the data set GSE107101 showed higher expression of YTHDF2 in advanced ICC tissues than in primary ICC tissues (Figure 1D). We then measured YTHDF2 levels in 96 paraffin‐embedded specimens from ICC patients using IHC assay (Figure 1E). YTHDF2 was significantly associated with certain clinicopathological characteristics, such as lymph node metastasis (Figure 1F), vascular invasion (Figure 1G) and tumour node metastasis (TNM) stage (Figure 1H). However, there were no correlations between YTHDF2 expression and other parameters such as sex, age, tumour size, cancer antigen 19‐9 (CA19‐9), total bilirubin and nerve invasion (Supplementary Table S2). The Kaplan–Meier analysis showed significantly lower OS (Figure 1I) and DFS (Figure 1J) of ICC patients with high YTHDF2 expression than that of those with low YTHDF2 expression. These results suggest that YTHDF2 might act as an oncogene in ICC.

### 3.2 Knockdown of YTHDF2 significantly inhibits ICC cell proliferation, promotes apoptosis and arrests cell cycle in G0/G1 stage

**FIGURE 2:**
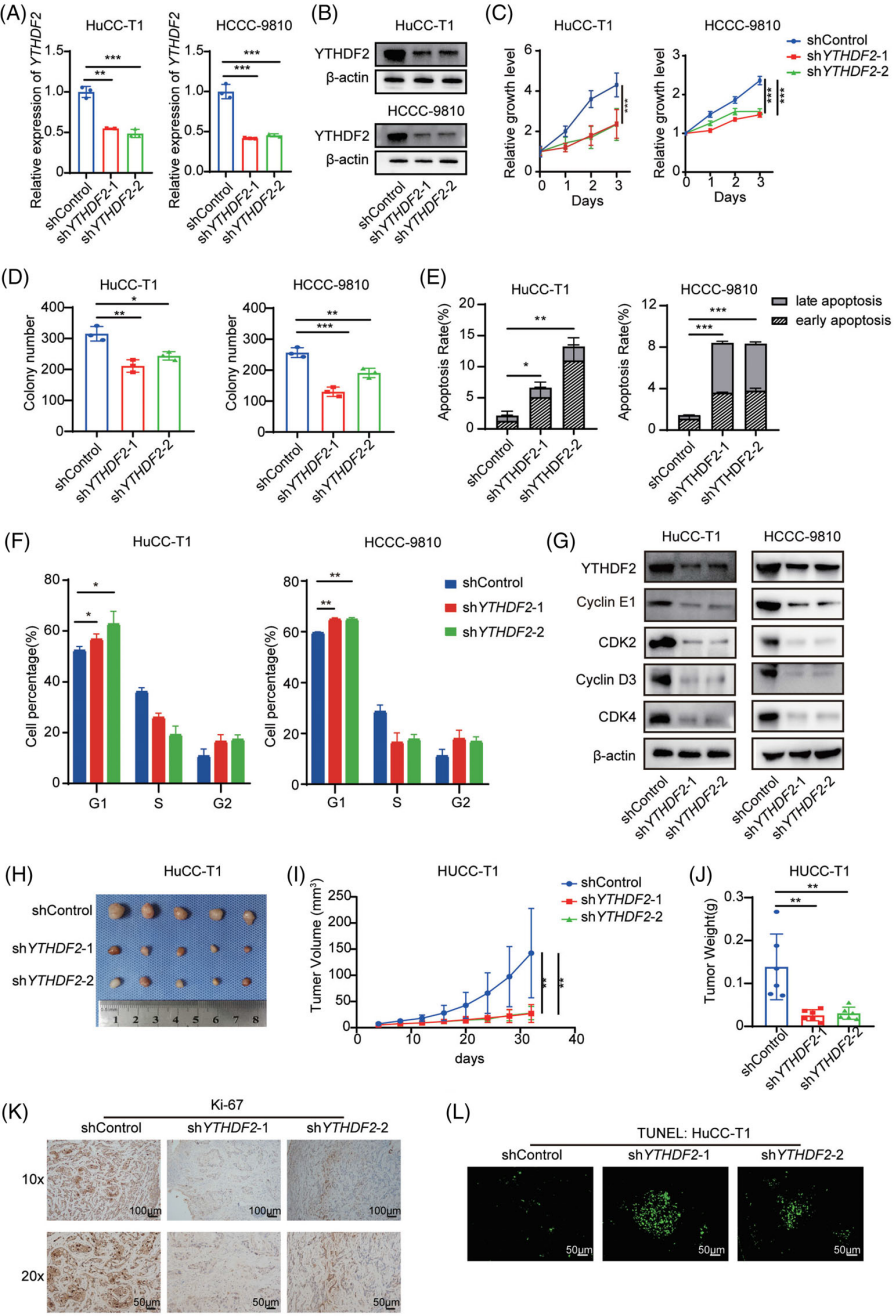
Knockdown of YTHDF2 significantly inhibits ICC cell proliferation, promotes apoptosis and arrests cell cycle in G0/G1 stage. (A) Relative mRNA expression of YTHDF2 in HuCC‐T1 and HCCC‐9810 cells transfected with shControl or sh*YTHDF2*. (B) The protein expression of YTHDF2 in HuCC‐T1 and HCCC‐9810 cells transfected with shControl or sh*YTHDF2* were measured by western blotting. (C) Cell viability of HuCC‐T1 and HCCC‐9810 cells transfected with shControl or sh*YTHDF2*. (D) Colony formation analysis of HuCC‐T1 and HCCC‐9810 cells transfected with shControl or sh*YTHDF2*. (E) Apoptosis analysis of HuCC‐T1 and HCCC‐9810 cells transfected with shControl or sh*YTHDF2*. (F) Cell cycle analysis of HuCC‐T1 and HCCC‐9810 cells transfected with shControl or sh*YTHDF2*. (G) Immunoblotting to measure YTHDF2, CDK2, cyclinD3, cyclinE1 and CDK4 protein levels in HuCC‐T1 and HCCC‐9810 cells transfected with shControl or sh*YTHDF2*. (H) The mice were executed 32 days post‐implantation of shControl or sh*YTHDF2*‐transfected HuCC‐T1 cells and the tumours from respective groups were photographed. (I) Tumour growth curves post‐implantation of shControl or sh*YTHDF2*‐ transfected HuCC‐T1 cells. Tumour volume was calculated every 4 days. (J) Tumour weight of each group was measured. (K) Representative IHC staining of Ki67 in tumours with different treatments. (L) Representative images of TUNEL analysis in tumours with different treatments. The data are presented as mean ± SD and compared by *t*‐test. *p < .05; **p < .01; *** *p* < .001

We next examined the mRNA expression of YTHDF2 in three human ICC cell lines (HuCC‐T1, HCCC‐9810 and RBE) and one normal intrahepatic bile duct cell line (HIBEC). YTHDF2 expression was higher in HuCC‐T1 and HCCC‐9810 than in RBE and HIBEC (Supplementary Figure S1A). To explore the function of YTHDF2 in ICC tumourigenesis, we then constructed stable YTHDF2 knockdown HuCC‐T1 and HCCC‐9810 cell lines using lentivirus carrying shRNA. Transfection efficiency was confirmed using qPCR (Figure 2A) and western blotting (Figure 2B). We then investigated the impact of YTHDF2 downregulation on cell proliferation using the CellTiter‐Glo Luminescent Cell Viability Assay and colony formation assay. Downregulation of YTHDF2 reduced the proliferation rate (Figure 2C) and colonies (Figure 2D and Supplementary Figure S1B) in both two ICC cell lines. Silencing YTHDF2 also significantly increased cell apoptosis (Figure 2E and Supplementary Figure S1C) and G0/G1 arrest (Figure 2F) in the two ICC cell lines. The results of western blotting revealed that YTHDF2 knockdown substantially reduced CDK2, cyclinD3, CDK4 and cyclinE1 protein levels (Figure 2G). YTHDF2 knockdown cells and control cells were further subcutaneously implanted into nude mice to verify the impact of YTHDF2 on ICC growth in vivo. YTHDF2 knockdown significantly inhibited tumour growth in HuCC‐T1 xenograft models (Figure 2H and I). Lower tumour weight was observed in the sh*YTHDF2*‐transfected HuCC‐T1 xenograft model when compared to that in the control group (Figure 2J). Moreover, IHC staining revealed that the proliferation marker gene Ki67 was markedly lower in YTHDF2 knockdown xenograft models (Figure 2K). The TUNEL assay results indicated that YTHDF2 knockdown groups had more apoptotic cells (Figure 2L).

### 3.3 Overexpression of YTHDF2 significantly promotes ICC cell proliferation, inhibits apoptosis and decreases cell cycle arrest in G0/G1 stage

**FIGURE 3:**
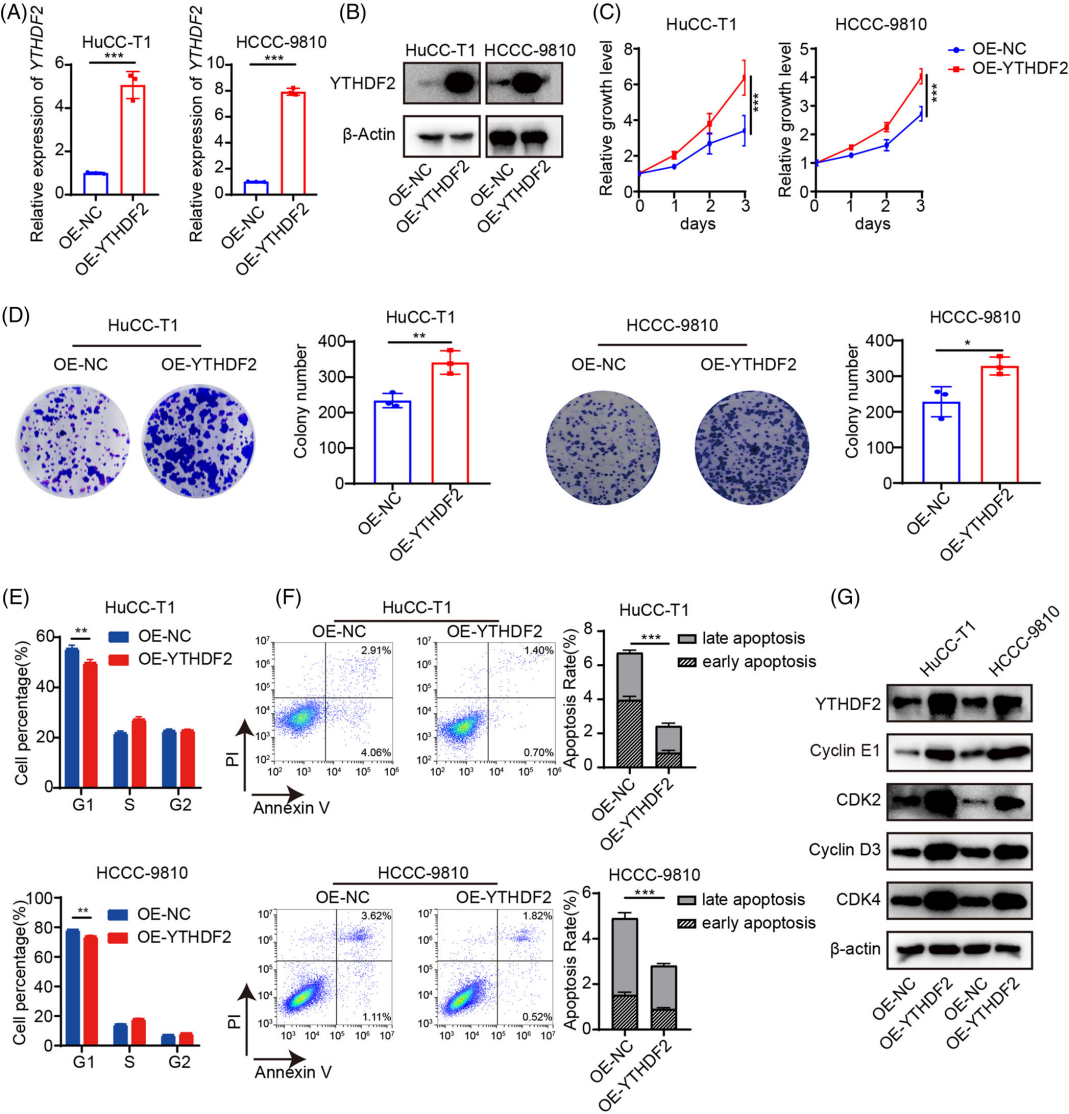
Overexpression of YTHDF2 significantly promotes ICC cell proliferation, inhibits apoptosis and decreases cell cycle arrest in G0/G1 stage. (A) The mRNA expression of YTHDF2 in HuCC‐T1 and HCCC‐9810 cells transfected with control or YTHDF2 vector were measured by qRT‐PCR. (B) The protein expression of YTHDF2 in HuCC‐T1 and HCCC‐9810 cells transfected with control or YTHDF2 vector were measured by western blotting. (C) Cell viability of HuCC‐T1 and HCCC‐9810 cells transfected with control or YTHDF2 vector. (D) Colony formation analysis of HuCC‐T1 and HCCC‐9810 cells transfected with control or YTHDF2 vector. (E) Cell cycle analysis of HuCC‐T1 and HCCC‐9810 cells transfected with control or YTHDF2 vector. (F) Apoptosis analysis of HuCC‐T1 and HCCC‐9810 cells transfected with control or YTHDF2 vector. (G) Immunoblotting to measure YTHDF2, CDK2, cyclinD3, cyclinE1 and CDK4 protein levels in HuCC‐T1 and HCCC‐9810 cells transfected with control or YTHDF2 vector. The data are presented as mean ± SD and compared by *t*‐test. *p < .05; **p < .01; *** *p* < .001

To test whether overexpression of YTHDF2 has the opposite effect on ICC cell proliferation, apoptosis and cell cycle, we constructed stable YTHDF2‐overexpressing HuCC‐T1 and HCCC‐9810 cell lines. The efficiency was confirmed using qPCR (Figure 3A) and western blotting (Figure 3B). Not surprisingly, YTHDF2 overexpression enhanced the proliferation (Figure 3C) and colony formation rates (Figure 3D) of HuCC‐T1 and HCCC‐9810 cells. Furthermore, YTHDF2 overexpression significantly reduced the duration of arrest of the G0/G1 stage (Figure 3E) in HuCC‐T1 and HCCC‐9810 cells, but had mild inhibitory effect on cell apoptosis (Figure 3F). Western blotting results showed that overexpression of YTHDF2 substantially increased CDK2, cyclinD3, CDK4 and cyclinE1 protein levels (Figure 3G). These findings suggest that YTHDF2 is involved in ICC carcinogenesis.

### 3.4 Overexpression of YTHDF2 desensitises ICC cells to cisplatin

**FIGURE 4:**
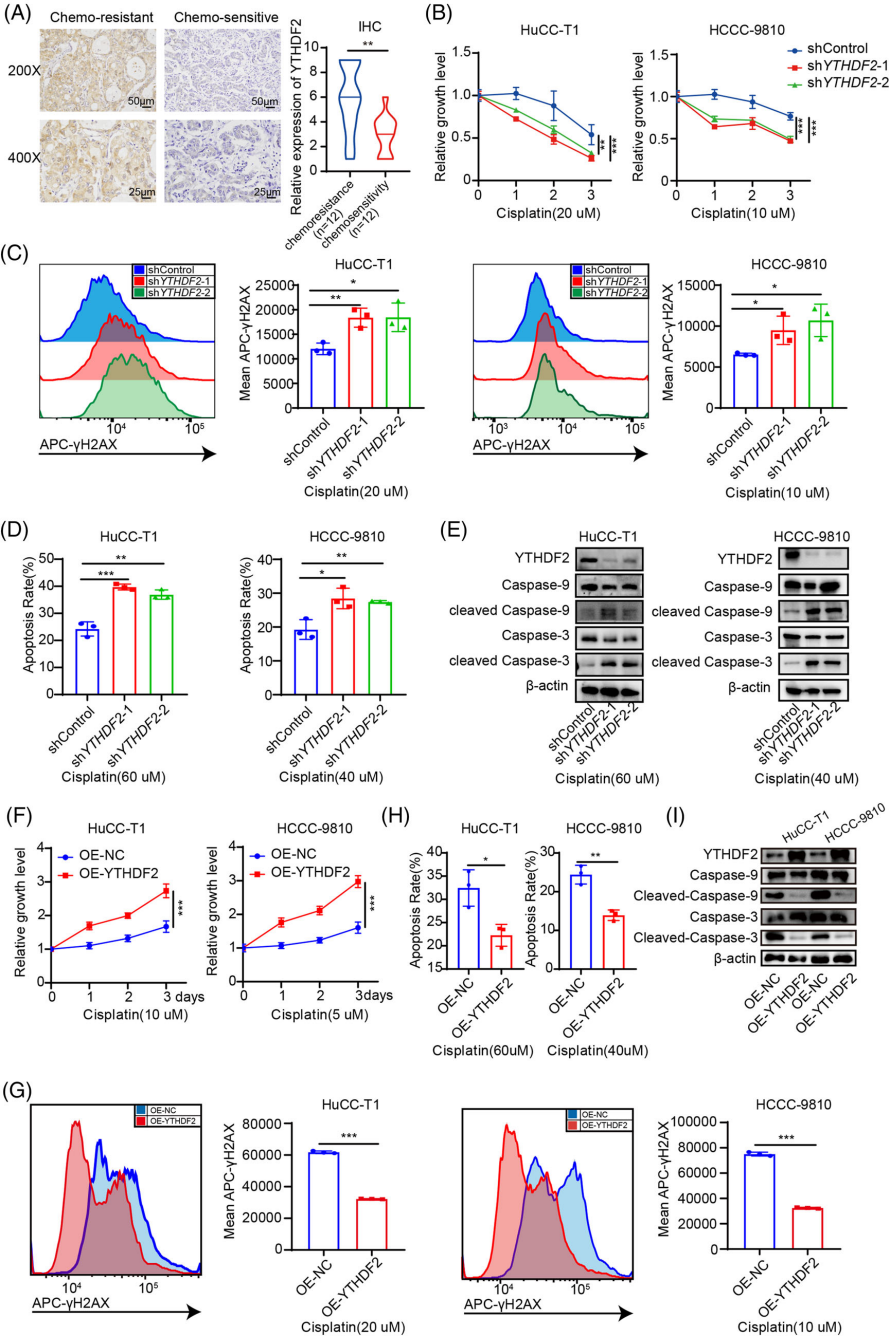
Overexpression of YTHDF2 desensitises ICC cells to cisplatin. (A) Representative images and the IHC scores for YTHDF2 in 12 chemoresisitant and 12 chemosensitive ICC tissues. (B) Cell viability of shControl or sh*YTHDF2*‐transfected HuCC‐T1 and HCCC‐9810 cells after treatment with cisplatin (20 or 10 μM, respectively). (C) Representative flow cytometry analysis and mean fluorescent intensity of γ‐H2AX in shControl or sh*YTHDF2*‐transfected HuCC‐T1 and HCCC‐9810 cells after treatment with cisplatin (20 or 10 μM, respectively). (D) Apoptosis analysis of shControl or sh*YTHDF2*‐transfected HuCC‐T1 and HCCC‐9810 cells after treatment with cisplatin (60 or 40 μM, respectively). (E) Immunoblotting to measure caspase‐9, cleaved caspase‐9, caspase‐3, cleaved caspase‐3 protein levels in shControl or sh*YTHDF2*‐transfected HuCC‐T1 and HCCC‐9810 cells after treatment with cisplatin (60 or 40 μM, respectively). (F) Cell viability of control or YTHDF2 vector‐transfected HuCC‐T1 and HCCC‐9810 cells after treatment with cisplatin (10 or 5 μM, respectively). (G) Representative flow cytometry analysis and mean fluorescent intensity of γ‐H2AX in control or YTHDF2 vector‐transfected HuCC‐T1 and HCCC‐9810 cells after treatment with cisplatin (20 or 10 μM, respectively). (H) Apoptosis analysis of control or YTHDF2 vector‐transfected HuCC‐T1 and HCCC‐9810 cells after treatment with cisplatin (60 or 40 μM, respectively). (I) Immunoblotting to measure caspase‐9, cleaved caspase‐9, caspase‐3, cleaved caspase‐3 protein levels in control or YTHDF2 vector‐transfected HuCC‐T1 and HCCC‐9810 cells after treatment with cisplatin (60 or 40 μM, respectively). The data are presented as mean ± SD and compared by *t*‐test. *p < .05; **p < .01; *** *p* < .001

To test the function of YTHDF2 in the cisplatin treatment of ICC, the YTHDF2 expression in 12 chemoresistant and 12 chemosensitive ICC tissues was detected by IHC. The results uncovered a higher expression of YTHDF2 in chemoresistant patients with ICC than in sensitive ones (Figure 4A). We then assessed changes in YTHDF2 expression after cisplatin treatment. We found that cisplatin treatment induced the upregulation of YTHDF2 in both a dose‐ and time‐dependent manner in HuCC‐T1 and HCCC‐9810 cells (Supplementary Figure S2A and Supplementary Figure S2B). As expected, downregulation of YTHDF2 can increase the sensitivity of HuCC‐T1 and HCCC‐9810 cells to cisplatin treatment (Figure 4B). Downregulation of YTHDF2 increased the DNA damage to cisplatin treatment in HuCC‐T1 and HCCC‐9810 cells (Figure 4C). Furthermore, YTHDF2 knockdown promoted cell apoptosis in response to cisplatin treatment in HuCC‐T1 and HCCC‐9810 cells (Figure 4D and Supplementary Figure S2C). Western blotting showed that YTHDF2 knockdown substantially increased the cleaved caspase 3 and cleaved caspase 9 protein levels following cisplatin administration in HuCC‐T1 and HCCC‐9810 cells (Figure 4E).

We then tested whether overexpression of YTHDF2 had the opposite effect to cisplatin treatment in ICC cells. As expected, overexpression of YTHDF2 desensitised HuCC‐T1 and HCCC‐9810 cells to cisplatin treatment (Figure 4F). Overexpression of YTHDF2 decreased the DNA damage to cisplatin treatment in HuCC‐T1 and HCCC‐9810 cells (Figure 4G). Furthermore, YTHDF2 overexpression inhibited cell apoptosis in response to cisplatin treatment in HuCC‐T1 and HCCC‐9810 cells (Figure 4H and Supplementary Figure S2D). Western blotting showed that YTHDF2 overexpression substantially decreased the cleaved caspase 3 and cleaved caspase 9 protein levels following cisplatin administration in HuCC‐T1 and HCCC‐9810 cells (Figure 4I). These findings suggest that YTHDF2 contributes to desensitisation against cisplatin in ICC.

### 3.5 CDKN1B is a downstream target of YTHDF2‐mediated m^6^A modification

**FIGURE 5:**
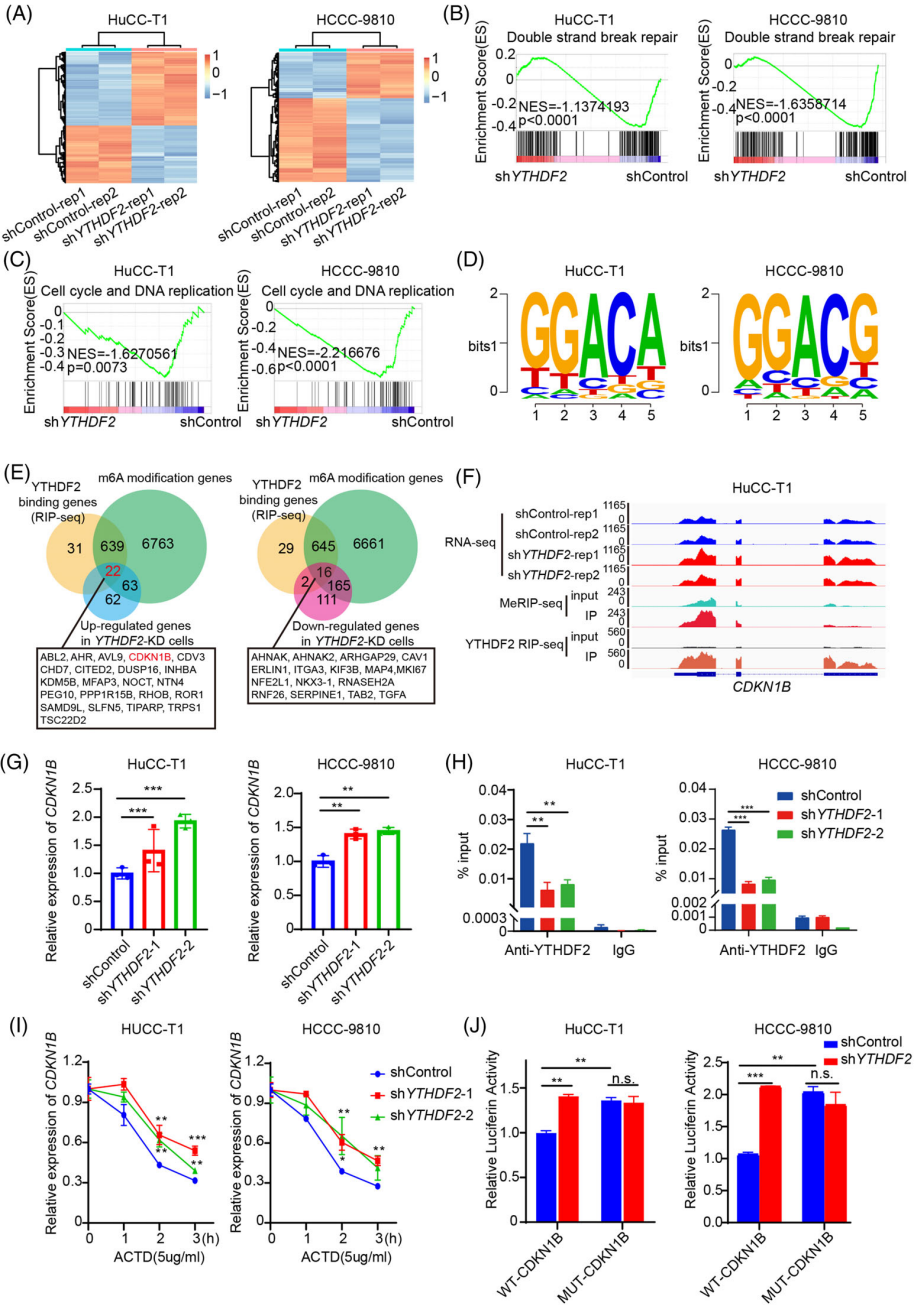
CDKN1B is one of the key downstream targets of YTHDF2. (A) The heatmap of RNA‐seq in control and YTHDF2‐deficient HuCC‐T1 and HCCC‐9810 cells. (B) The GSEA revealed that DNA double strand break repair were enriched in HuCC‐T1 and HCCC‐9810 cells transfected with sh*YTHDF2*. (C) The GSEA revealed that Cell cycle and DNA replication were enriched in HuCC‐T1 and HCCC‐9810 cells transfected with sh*YTHDF2*. (D) The motifs of m6A peaks are RRACH (R = G/A, H = A/C/U). (E) Venn diagram showing the overlap of genes differentially expressed in HuCC‐T1 and HCCC‐9810 cells transfected with sh*YTHDF2* and containing m6A and YTHDF2 binding sites. (F) Integrative genomics viewer (IGV) plots showed the m6A binding site and YTHDF2 binding site and the change of RNA‐seq peaks at *CDKN1B* mRNAs in HuCC‐T1. (G) The mRNA expression of CDKN1B in HuCC‐T1 and HCCC‐9810 cells transfected with shControl or sh*YTHDF2* were measured by qRT‐PCR. (H) RIP‐qPCR assay using YTHDF2‐specific antibody and IgG control antibody to detect the enrichment of YTHDF2 binding to CDKN1B m6A modification sites. (I) qRT‐PCR analysis of the decay rate of CDKN1B mRNA at the indicated times after actinomycin D (5 μg/ml) treatment in HuCC‐T1 and HCCC‐9810 cells with or without YTHDF2 silencing. (J) Relative luciferase activity of CDKN1B 3’UTR with wild‐type or mutated m^6^A sites after YTHDF2 silencing in HuCC‐T1 and HCCC‐9810 cells. Renilla luciferase activity was measured and normalised to firefly luciferase activity. The data are presented as mean ± SD and compared by *t*‐test. *p < .05; **p < .01; *** *p* < .001

We subsequently performed RNA‐seq for the control and YTHDF2‐deficient HuCC‐T1 and HCCC‐9810 cells (Figure 5A). Through the DEseq package in R language, a total of 1399 and 1301 differentially expressed genes (DEGs) were identified in YTHDF2‐KD HuCC‐T1 cells (788 genes upregulated and 611 genes downregulated) and YTHDF2‐KD HCCC‐9810 cells (458 genes upregulated and 843 genes downregulated) (Supplementary Figure S3A). Gene set enrichment analysis (GSEA) showed that the expression of genes associated with double‐strand break repair (Figure 5B) and cell cycle DNA replication (Figure 5C) significantly decreased in the YTHDF2 knockdown group compared with control group. Further, MeRIP‐seq was performed in HuCC‐T1 and HCCC‐9810 cells. The motifs of the m^6^A peaks were consistent with the consensus sequence (RRACH (R = G/A, H = A/C/U)) (Figure 5D). The distribution pattern analyses suggested that the peaks were highly enriched in the start and stop codon regions (Supplementary Figure S3B and S3C). The major function of YTHDF2 is mediating target mRNA degradation by recognising and binding to m^6^A modification sites of target mRNAs. Therefore, we performed an anti‐YTHDF2 RIP‐seq. There were 22 genes containing m^6^A and YTHDF2 binding sites that were upregulated upon YTHDF2 knockdown (Figure 5E). The expression of m^6^A‐modified YTHDF2 binding genes in the YTHDF2 knockdown group were higher than those in the control group (Supplementary Figure S3D). Through a literature review, we found that CDKN1B was reported as a tumour suppressor gene associated with drug resistance. Integrative genomics viewer plots demonstrated the m^6^A modification sites, the YTHDF2 binding sites and the change in RNA‐seq peaks of *CDKN1B* mRNAs upon YTHDF2 knockdown in HuCC‐T1 cells (Figure 5F) and HCCC‐9810 cells (Supplementary Figure S3E). qRT‐PCR confirmed that the *CDKN1B* mRNA levels were higher in both HuCC‐T1 and HCCC‐9810 cells with stable YTHDF2 knockdown than in vector cells (Figure 5G). We further measured YTHDF2 levels in the 96 paraffin‐embedded specimens using IHC assay (Supplementary Figure S4A). The expression of CDKN1B was negatively correlated with the expression of YTHDF2 (Supplementary Figure S4B). Kaplan–Meier analysis showed significant poorer OS (Supplementary Figure S4C) and DFS (Supplementary Figure S4D) in ICC patients with low CDKN1B expression than that in those with high CDKN1B expression.

METTL3 is the core component of m^6^A methyltransferases complex. We further conducted MeRIP‐qPCR to evaluate the m^6^A levels on CDKN1B mRNA upon silencing METTL3 or not. The result showed that the m^6^A levels on CDKN1B mRNA also reduced upon silencing METTL3 (Supplementary Figure S5A). YTHDF2 RIP‐qPCR was also conducted to evaluate the binding of YTHDF2 to CDKN1B mRNA upon METTL3 or YTHDF2 knockdown. The results showed that both the enrichment of YTHDF2 to CDKN1B mRNA reduced upon METTL3 knockdown (Supplementary Figure S5B) or YTHDF2 knockdown (Figure 5H). RNA stability assays showed that knockdown of YTHDF2 decelerated the mRNA decay of CDKN1B (Figure 5I). We then constructed a wild‐type (WT) CDKN1B and an m^6^A site mutated CDKN1B dual luciferase reporter. A dual luciferase activity assay showed that the luciferase activity of the WT CDKN1B dual luciferase reporter was increased upon YTHDF2 knockdown, while the luciferase activity of the m^6^A site mutated luciferase reporter did not differ significantly (Figure 5J). These results indicate that CDKN1B is a downstream gene of YTHDF2, and that YTHDF2 promotes the degradation of CDKN1B mRNA in an m^6^A‐dependent manner.

### 3.6 YTHDF2 facilitates ICC progression and cisplatin insensitivity by downregulating CDKN1B expression

**FIGURE 6:**
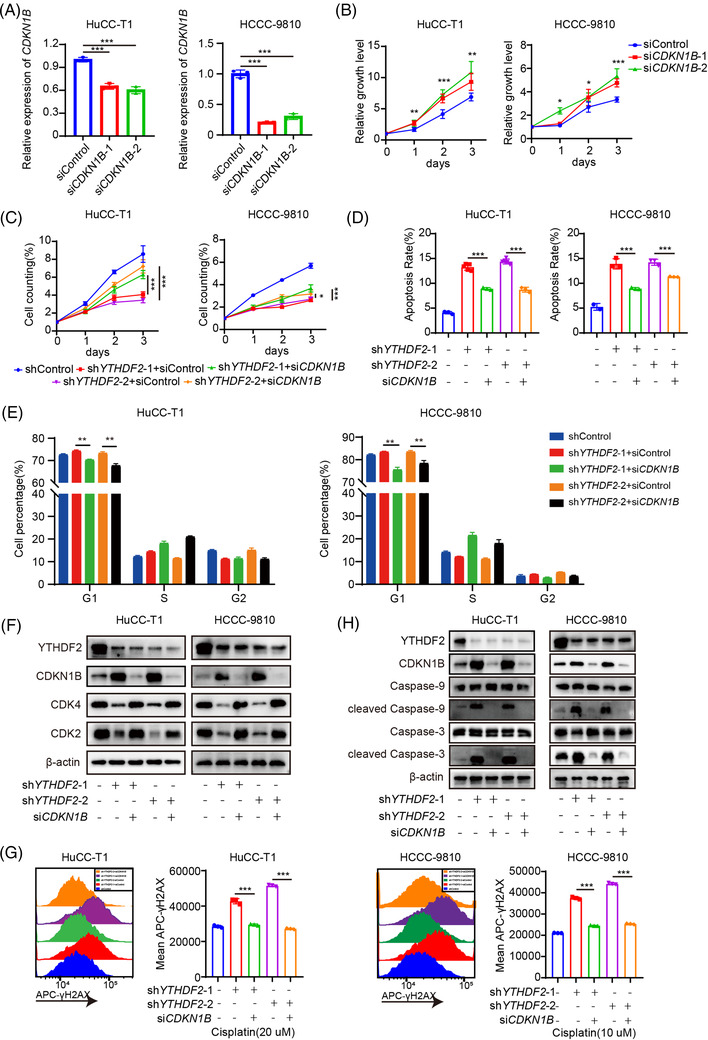
YTHDF2 facilitates the ICC progression and cisplatin resistance by modulating CDKN1B mRNA decay. (A) Relative mRNA expression of CDKN1B in HuCC‐T1 and HCCC‐9810 cells transfected with siControl or si*CDKN1B* for 48 h. (B) Cell viability of HuCC‐T1 and HCCC‐9810 cells transfected with siControl or si*CDKN1B* for 48 h. (C) Cell viability of HuCC‐T1 and HCCC‐9810 cells co‐transfected with indicated shRNA or/and siRNA for 48 h. (D) Apoptosis analysis of HuCC‐T1 and HCCC‐9810 cells co‐transfected with indicated shRNA or/and siRNA for 48 h. (E) Cell cycle analysis of HuCC‐T1 and HCCC‐9810 cells co‐transfected with indicated shRNA or/and siRNA for 48 h. (F) Immunoblotting to measure CDK2 and CDK4 protein levels in HuCC‐T1 and HCCC‐9810 cells co‐transfected with indicated shRNA or/and siRNA for 48 h. (G) Representative flow cytometry analysis and mean fluorescent intensity of γ‐H2AX in indicated shRNA or/and siRNA co‐transfected HuCC‐T1 and HCCC‐9810 cells after treatment with cisplatin (20 or 10 μM, respectively). (H) Immunoblotting to measure caspase‐9, cleaved caspase‐9, caspase‐3, cleaved caspase‐3 protein levels in indicated shRNA or/and siRNA co‐transfected HuCC‐T1 and HCCC‐9810 cells after treatment with cisplatin (60 or 40 μM, respectively). The data are presented as mean ± SD and compared by *t*‐test. *p < .05; **p < .01; *** *p* < .001

Two siRNAs targeting CDKN1B was designed and the knockdown efficiency was verified using qRT‐PCR (Figure 6A). CDKN1B knockdown dramatically promoted cell proliferation (Figure 6B), inhibited apoptosis (Supplementary Figure S6A), and reduced the duration of the G0/G1 stage (Supplementary Figure S6B) in both HuCC‐T1 and HCCC‐9810 cells. Then, CDKN1B expression was knocked down in stable YTHDF2‐KD ICC cells using siRNAs targeting CDKN1B. As expected, CDKN1B knockdown rescued the proliferation (Figure 6C), apoptosis (Figure 6D and Supplementary Figure S6C) and cell cycle (Figure 6E) of YTHDF2 knockdown ICC cells. Western blotting showed that the knockdown of CDKN1B in YTHDF2 knockdown ICC cells increased the proteins expression of CDK2 and CDK4 (Figure 6F). Furthermore, the knockdown of CDKN1B rescued the DNA damage induced by cisplatin treatment in YTHDF2 knockdown ICC cells (Figure 6G). Western blotting showed that knockdown of CDKN1B in YTHDF2 knockdown ICC cells rescued the protein expression of cleaved caspase 3 and cleaved caspase 9 (Figure 6H).

We then perform experiments to detect whether OE‐CDKN1B can rescue the pro‐oncogenic effect of OE‐YTHDF2 in ICC. The results showed that CDKN1B overexpression rescued the proliferation (Supplementary Figure S7A), apoptosis (Supplementary Figure S7B) and cell cycle (Supplementary Figure S7C) of YTHDF2 overexpression ICC cells among HuCC‐T1 and HCCC‐9810 cells. Furthermore, the overexpression of CDKN1B rescued the DNA damage induced by cisplatin treatment in YTHDF2 overexpression HuCC‐T1 and HCCC‐9810 cells (Supplementary Figure S7D). Collectively, these observations indicate that YTHDF2 inhibits apoptosis, accelerates the cell cycle and desensitises ICC to cisplatin treatment by downregulating the expression of CDKN1B in an m^6^A‐dependent manner.

### 3.7 Combination treatment of siYTHDF2 and cisplatin enhances the anti‐tumour effect of cisplatin in a chemoresistant PDX model of ICC

**FIGURE 7:**
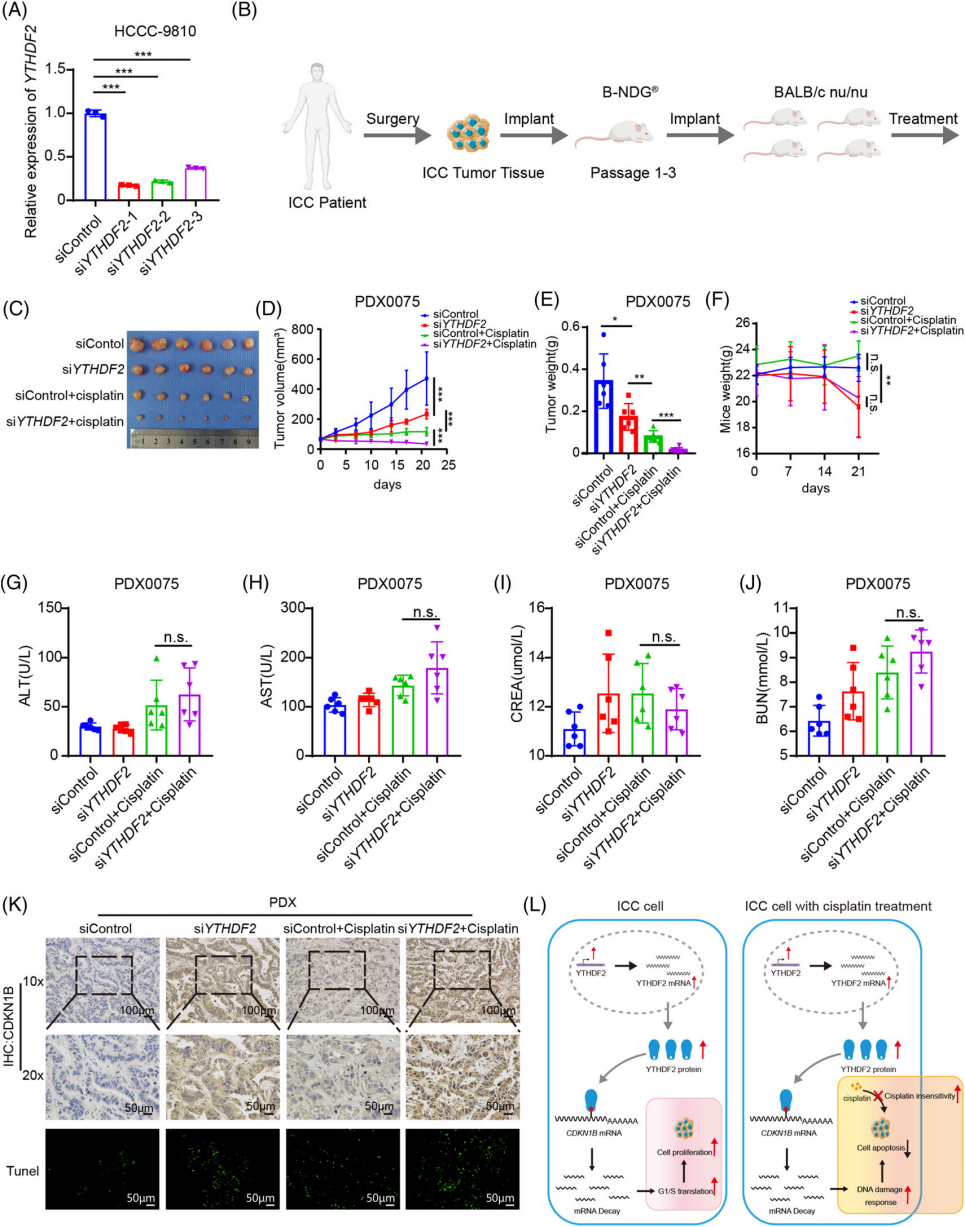
Combination of siYTHDF2 and cisplatin enhanced the anti‐tumour effect of cisplatin in a chemoresistant PDX model of ICC. (A) The mRNA expression of YTHDF2 in HuCC‐T1 and HCCC‐9810 cells transfected with siControl or si*YTHDF2* were measured by qRT‐PCR. (B) A graphic illustration of the construction of ICC PDX model. (C) Mice bearing passage 3 tumour of PDX0075 were randomly divided into four groups and treated with siControl (3nmol per mouse, twice a week), si*YTHDF2* (3 nmol per mouse, twice a week), siControl plus cisplatin (siControl, 3 nmol per mouse, twice a week and cisplatin, 4 mg/kg, once a week) or si*YTHDF2* plus cisplatin (si*YTHDF2*, 3 nmol per mouse, twice a week and cisplatin, 4 mg/kg, once a week) for 21 days, then were executed and photographed. (D) Tumour growth curves of each group were shown. Tumour volume was calculated twice a week. (E) Tumour weight of each group was measured. (F) Body weight of the mice was measured once a week. (G) Serum ALT of each group was measured. (H) Serum AST of each group was measured. (I) Serum CREA of each group was measured. (J) Serum BUN of each group was measured. (K) Representative IHC staining of CDKN1B and representative image of TUNEL in tumours with different treatments. (L) The graphical abstract illustrated that YTHDF2 recognised the m^6^A site on *CDKN1B* mRNA and accelerates its mRNA decay, which promotes tumourigenesis via accelerating cell cycle and desensitises ICC to cisplatin treatment via decreasing DNA damage, and then inhibiting apoptosis. The data are presented as mean ± SD and compared by *t*‐test. *p < .05; **p < .01; *** *p* < .001

Based on the effect of YTHDF2 on ICC tumourigenesis and desensitisation to cisplatin, we further explored the effect of combination treatment with siYTHDF2 and cisplatin in a chemoresistant PDX model of ICC (PDX0075) from a patient who relapsed in 6 months after R0 resection and subsequent chemotherapy with cisplatin and gemcitabine. First, we tested the knockdown efficiency of siRNA targeting YTHDF2 using qRT‐PCR and found that YTHDF2 mRNA expression decreased by more than 80% with siYTHDF2 #1 (Figure 7A). siYTHDF2 #1 was then used in subsequent experiments. The third passage tumours of PDX0075 were implanted into 5‐week‐old female BALB/c nude mice (Figure 7B). Once the xenografts reached a volume of ∼50 mm^3^, the PDX‐bearing mice were randomly divided into four groups, and then injected with 3 nmol siRNA intratumourally twice a week and/or intraperitoneal injected with cisplatin (4 mg/kg) once a week for 21 days. Notably, treatment with both siYTHDF2 #1 and cisplatin significantly reduced the tumour volume (Figure 7C and D) and weight (Figure 7E) compared to treatment with cisplatin alone. Moreover, the combined treatment did not cause any significant difference in mice weight (Figure 7F), liver toxicity (Figure 7G and H) and kidney toxicity (Figure 7I and J) when compared with that in cisplatin‐treated mice. IHC staining revealed that the proliferation marker gene Ki67 was least expressed in the combination treatment group (Supplementary Figure S8) and the expression of CDKN1B was highest in the combination treatment group (Figure 7K). The TUNEL assay showed that the highest number of apoptotic cells presented in the combination treatment group (Figure 7K). Altogether, these results show that YTHDF2 downregulation enhances the anti‐tumour effect of cisplatin via both proliferation inhibition and sensitisation of ICC cells to cisplatin treatment (Figure 7L).

## 4 DISCUSSION

ICC is one of the most aggressive types of cancer. For ICC patients with locally advanced or metastatic disease, no effective treatment is available, and overall survival remains poor despite of advances in the development of new chemotherapy and targeted therapies. Epitranscriptomic regulation of ICC has been reported to play an important role in tumourigenesis and chemoresistance. METTL3, the core component of m^6^A methyltransferase, promotes ICC proliferation, invasion and metastasis via downregulating IFIT2 expression.^21^ FTO, a demethylase of m^6^A modification, promotes ICC progression via impairing the mRNA stability of TEAD2.^22^ Another m^6^A demethylase ALKBH5 orchestrates PD‐L1 expression and regulates immunotherapy efficacy.^23^ However, the role of YTHDF2 in oncogenesis and cisplatin treatment of ICC has not been investigated. In the present study, we revealed that YTHDF2 exerts important role in tumourigenesis and cisplatin‐desensitising function of ICC. Mechanistically, YTHDF2 recognised the m^6^A site on *CDKN1B* mRNA and accelerated its mRNA decay. The decreased expression of CDKN1B then promotes tumourigenesis by accelerating the cell cycle and further desensitises ICC to cisplatin treatment by decreasing DNA damage and inhibiting apoptosis.

YTHDF2, an important ‘reader’ protein of m^6^A modification, exhibits a series of biological processes involved in cancer carcinogenesis, including proliferation, invasion and metastasis. Zhou et al. reported that YTHDF2 contributed to the aberrant m^6^A modification of tripartite motif containing 7 (TRIM7), which regulates tumourigenesis in osteosarcoma.^24^ Zhang et al. revealed that YTHDF2 promoted lung cancer metastasis by modulating the m^6^A methylation of octamer‐binding transcription factor 4 (OCT4) mRNA.^25^ Further, YTHDF2 was dysregulated and played an oncogenic role in bladder cancer, hepatocellular carcinoma, cervical cancer, gastric cancer and pancreatic cancer.^26^ However, the role of YTHDF2 in ICC tumourigenesis has not yet been investigated. Our study implied that YTHDF2 plays a critical role in accelerating the cell cycle from G1 to S stage via the activation of cyclin E‐CDK2 or cyclin D‐CDK4 complexes, thereby promoting the proliferation of ICC.

The resistance to chemotherapy has been reported to be influenced by m^6^A modification. FTO‐β‐catenin‐ERCC1 axis can enhance the chemoradiotherapy resistance in cervical squamous cell carcinoma.^27^ METTL3 can promote chemo‐ and radioresistance in human pancreatic cancer.^28^ The overexpression of ALKBH5 can sensitise pancreatic cancer to chemotherapy, which is dependent on enhancing the expression of WIF‐1 and activating Wnt signalling.^29^ Cisplatin is one of the most widely used anticancer drugs in various tumours. Cisplatin‐based combination therapy has become the first‐line treatment for advanced or metastatic ICC patients. Cisplatin insensitivity and resistance have been partly attributed to the dysregulation of m^6^A modification. However, their roles are diverse. For example, methyltransferase‐like 3 (METTL3) could stabilise the transcription factor AP‐2 gamma (TFAP2C) mRNA in an insulin‐like growth factor 2 mRNA binding protein 1 (IGF2BP1)‐dependent mode and further enhance cisplatin resistance in seminoma.^30^ However, METTL3 promotes ferroptosis suppressor protein 1 (FSP1)‐mediated ferroptosis and leads to cisplatin sensitivity in non‐small cell lung carcinoma.^31^ Another m^6^A methyltransferase, Wilms tumour 1 associated protein (WTAP), contributes to cisplatin resistance by stabilising dual specificity phosphatase 6 (DUSP6) mRNA in an m^6^A‐dependent manner in nasal‐type natural killer/T‐cell lymphoma.^32^ The role of YTHDF2 in ICC cisplatin treatment has not yet been investigated. The results of our study revealed that YTHDF2 is upregulated in chemoresistant ICC tissues. Mechanistically, the upregulation of YTHDF2 can inhibit apoptosis by inhibiting the DNA damage and desensitises ICC to cisplatin treatment.

The *CDKN1B* gene encodes a cyclin‐dependent kinase inhibitor, p27. The encoded protein can bind to and inhibit the activation of cyclin D‐CDK4 or cyclin E‐CDK2 complexes, and thus arrest the cell cycle at G1 stage.^33^ Downregulation of p27 may be associated with poor prognosis in a variety of cancers, including ICC.^34^, ^35^ Cisplatin is a DNA alkylating agent, and its cytotoxic effects are attributable to DNA damage.^36^ Therefore, a decreased DNA damage and potentiated DNA repair may contribute greatly to cisplatin insensitivity and resistance.^37^ Emerging evidence has shown that p27 can enhance the DNA damage response^38^, ^39^; however, the role of p27 in cisplatin treatment is still unknown. In our study, we identified *CDKN1B* as the downstream gene of YTHDF2 through an integrated analysis using RNA‐seq, MeRIP‐seq and anti‐YTHDF2 RIP‐seq. Gain‐of‐function assays showed that YTHDF2 facilitates ICC progression and desensitises ICC to cisplatin treatment by downregulating CDKN1B expression. YTHDF2 mainly accelerates the mRNA decay of target genes. In our study, we found that YTHDF2 accelerates the degradation of *CDKN1B* mRNA in an m^6^A‐dependent manner. Therefore, based on these results, we propose that YTHDF2 can both promote ICC tumourigenesis and desensitise ICC to cisplatin treatment. This was also confirmed in a chemoresistant PDX model of ICC with combination treatment of *YTHDF2* siRNA and cisplatin (Supplementary Figure S9). Since the approval of the first siRNA therapeutic, ONPATTRO™ (Patisiran), for the treatment of transthyretin‐mediated amyloidosis,^40^ more and more siRNA therapeutics are under clinical trials for diverse diseases, especially cancers.^41^ Combination therapy with siRNAs and chemotherapy, which may overcome resistance, generate synergistic anti‐tumour effects and reduce side effect, is a promising treatment.^42^ Nanoparticle delivery systems combining YTHDF*2* siRNA and cisplatin may be an effective therapeutic for ICC treatment.

## 5 CONCLUSIONS

In conclusion, our findings demonstrate that overexpression of YTHDF2 promotes proliferation, inhibits apoptosis, decreases the arrest of the G0/G1 stage and desensitises ICC cells to cisplatin, which is dependent on the degradation of *CDKN1B* mRNA in an m^6^A‐dependent manner. These findings revealed the importance of the m^6^A modification in ICC and provided new insight in improving the efficacy of cisplatin in the future.

## CONFLICT OF INTEREST

No potential conflict of interest to disclose.

## Supporting information

Supporting Information
