# Supplementary material for: YTHDF2 promotes intrahepatic cholangiocarcinoma progression and desensitises cisplatin treatment by increasing CDKN1B mRNA degradation
Source: Clin Transl Med. 2022 Jun 13;12(6):e848. doi: 10.1002/ctm2.848 (PMC9191870; doi:10.1002/ctm2.848)
Supplement: Supplementary file 1 — Supporting Information [file CTM2-12-e848-s001.pdf]

Supplementary Materials

YTHDF2 promotes intrahepatic cholangiocarcinoma progression and desensitizes cisplatin treatment by increasing CDKN1B mRNA degradation

Chen-Song Huang[1#], Ying-Qin Zhu[1#], Qiong-Cong Xu[1#], Siyun Chen[2], Yue Huang[3], Guangyin Zhao[4], Xuhao Ni[1], Bo Liu[2], Wei Zhao[2]* and Xiao-Yu Yin[1]*

Contents

Supplemental Figure 1-9

Supplemental Table 1-2

Supplemental Methods

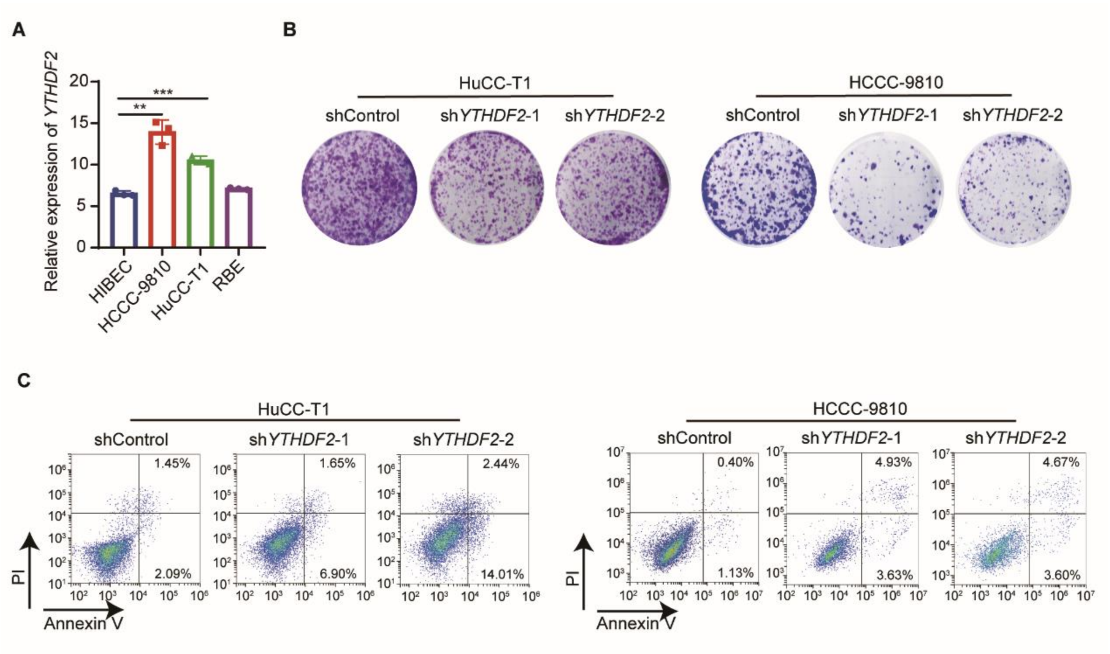

Figure S1. Knockdown of YTHDF2 significantly inhibits ICC cell proliferation, promotes apoptosis and arrests cell cycle in G0/G1 stage

A. Expression levels of YTHDF2 in ICC cell lines and normal intrahepatic bile duct cell.

B. Representative images of colony-forming assays of HuCC-T1 and HCCC-9810 cells transfected with shControl or sh*YTHDF2*.

C. Representative images of apoptosis analysis of HuCC-T1 and HCCC-9810 cells transfected with shControl or sh*YTHDF2*.

The data are presented as mean ± SD and compared by t-test. **$P < 0.01$; *** $P < 0.001$.

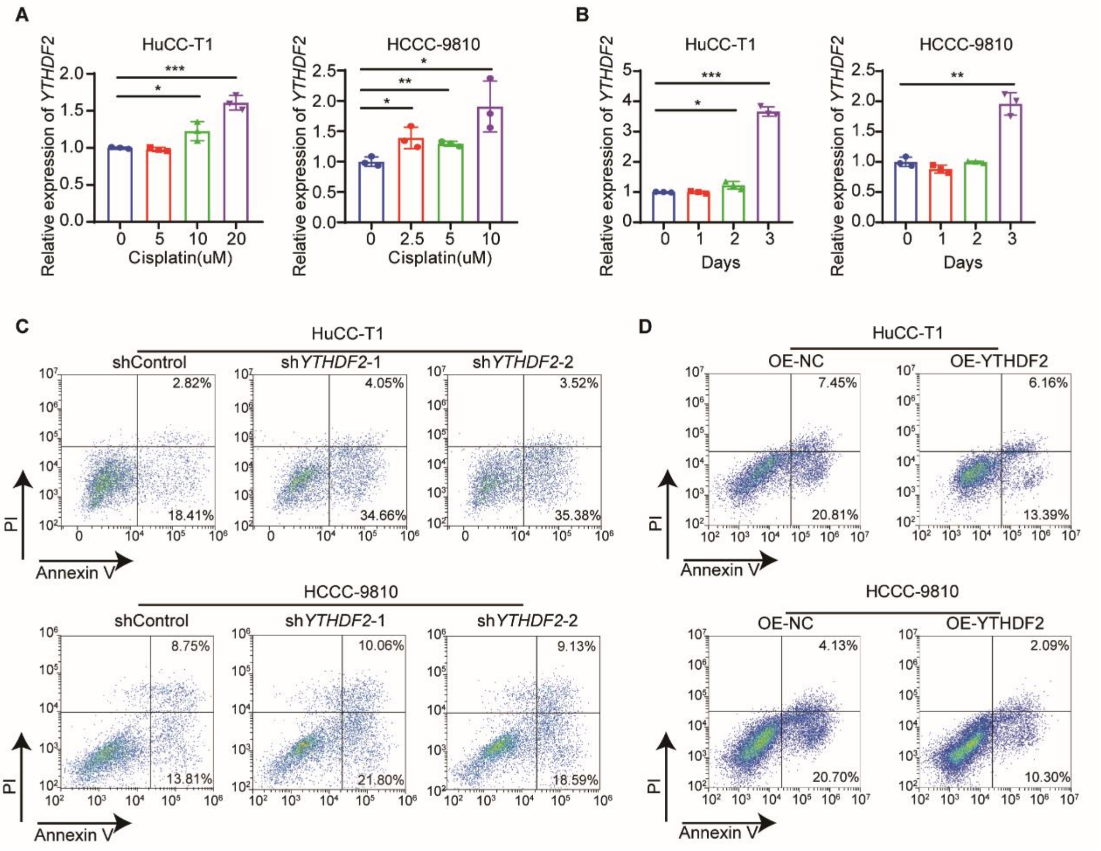

Figure S2. Overexpression of YTHDF2 desensitizes ICC cells to cisplatin.

A. Relative mRNA expression of YTHDF2 in HuCC-T1 and HCCC-9810 cells after different dose of cisplatin treatment for 48 hours.

B. Relative mRNA expression of YTHDF2 at indicated time points in HuCC-T1 and HCCC-9810 cells after treatment with cisplatin (20 μM or 10 μM, respectively).

C. Representative images of apoptosis analysis of shControl or sh*YTHDF2*-transfected HuCC-T1 and HCCC-9810 cells after treatment with cisplatin (60 μM or 40 μM, respectively).

D. Representative images of apoptosis analysis of control or YTHDF2 vector-transfected HuCC-T1 and HCCC-9810 cells after treatment with cisplatin (60 μM or 40 μM, respectively).

The data are presented as mean ± SD and compared by t-test. **P* < 0.05; ***P* < 0.01;

*** $P < 0.001$.

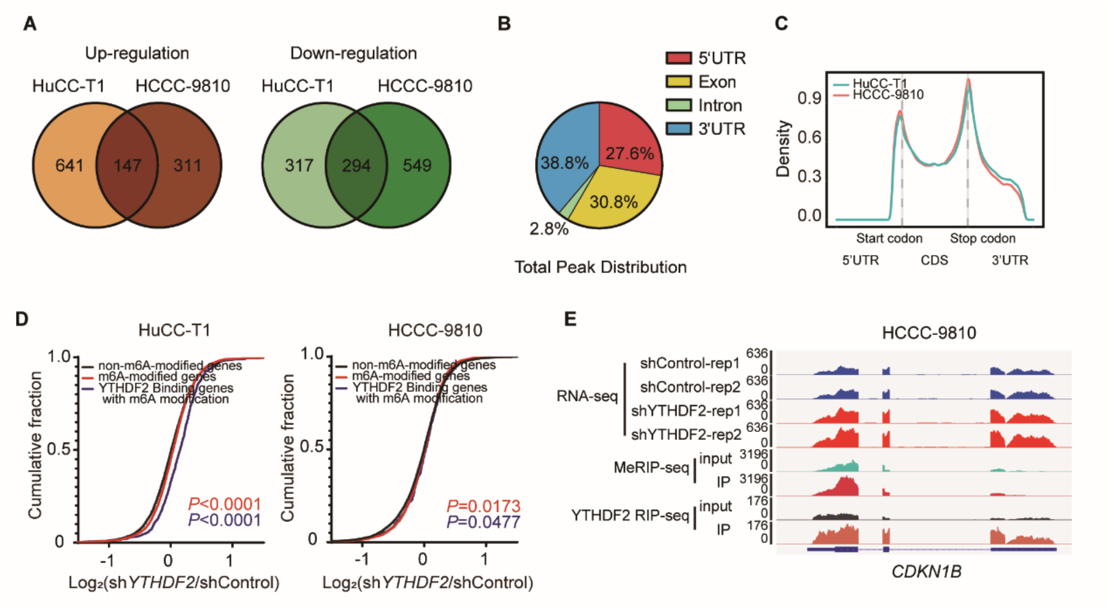

Figure S3. CDKN1B is a downstream target of YTHDF2-mediated m^{6}A modification

A. The venn diagram of differentially expressed genes (DEGs) in HuCC-T1 and HCCC-9810 cells after YTHDF2 knockdown.

B-C. Distribution of m^{6}A modification peak reads across all mRNAs in HuCC-T1 and HCCC-9810 cells.

D. Cumulative distribution of mRNA $\log_2$ fold changes between YTHDF2 knockdown and control for non-m^{6}A modified transcripts (black), m^{6}A modified transcripts (red), and YTHDF2 binding transcripts with m^{6}A modification (blue). *P* value were calculated using two-sided Mann-Whitney.

E. Integrative genomics viewer (IGV) plots of RNA-seq peaks, MeRIP-seq peaks and YTHDF2 RIP-seq peak at *CDKN1B* mRNAs in HCCC-9810 cells.

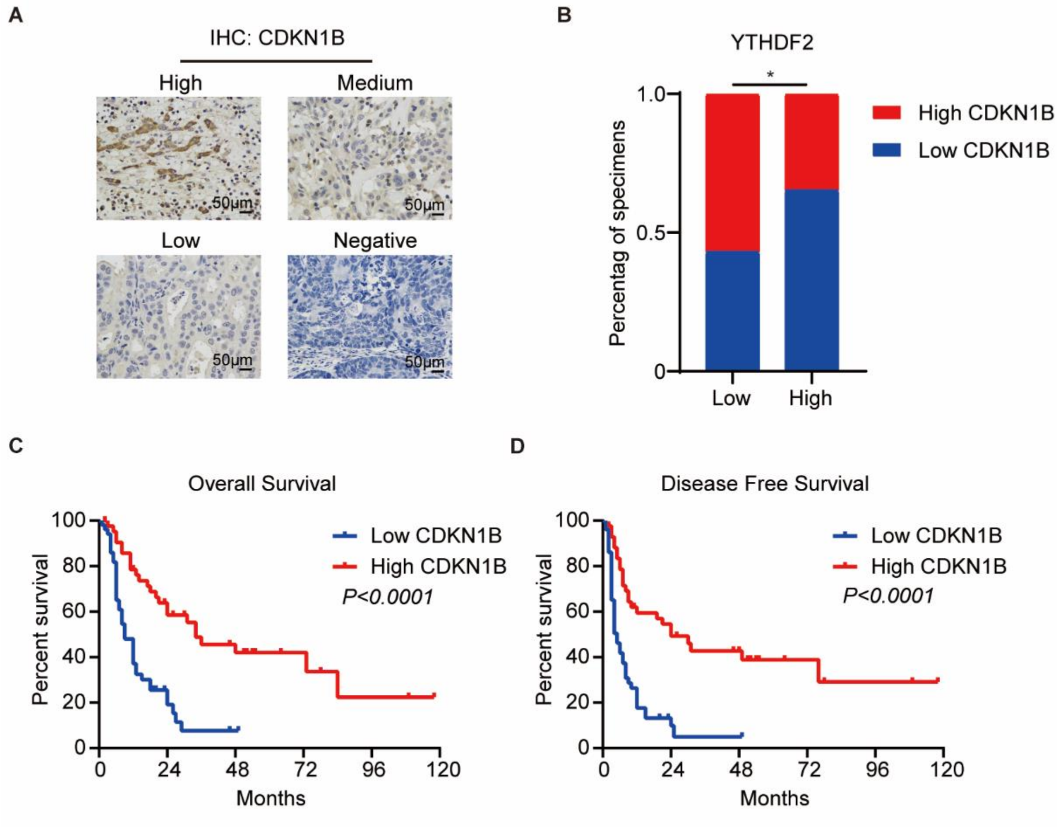

Figure S4. Low expression of CDKN1B is correlated with poor prognosis of ICC patients.

A. Representative images of IHC staining for CDKN1B in ICC tumors expressing low or high levels of CDKN1B.

B. Bar graphs indicate the correlation of YTHDF2 expression with CDKN1B expression in ICC specimens.

C. Kaplan–Meier survival curves of OS in 96 patients with ICC. (CDKN1B low expression, n =53 vs. CDKN1B high expression, n = 43). The *P* value was calculated using the log-rank test. HR hazard ratio.

D. Kaplan–Meier survival curves of DFS in 96 patients with ICC. (CDKN1B low expression, n = 53 vs. CDKN1B high expression, n = 43). The *P* value was calculated using the log-rank test. HR hazard ratio.

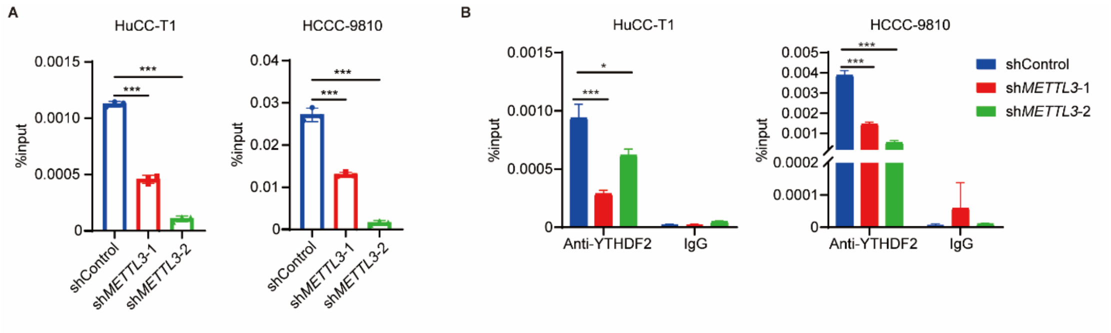

Figure S5. CDKN1B is regulated by YTHDF2 in an METTL3 dependent manner.

A. MeRIP-qPCR analysis of the m^6A levels on CDKN1B mRNA upon silencing METTL3 or not.

B. RIP-qPCR assay using YTHDF2-specific antibody and IgG control antibody to detect the enrichment of YTHDF2 binding to CDKN1B m^6A modification sites upon silencing METTL3 or not.

The data are presented as mean ± SD and compared by t-test. $*P < 0.05$; $**P < 0.01$; $***\ P < 0.001$.

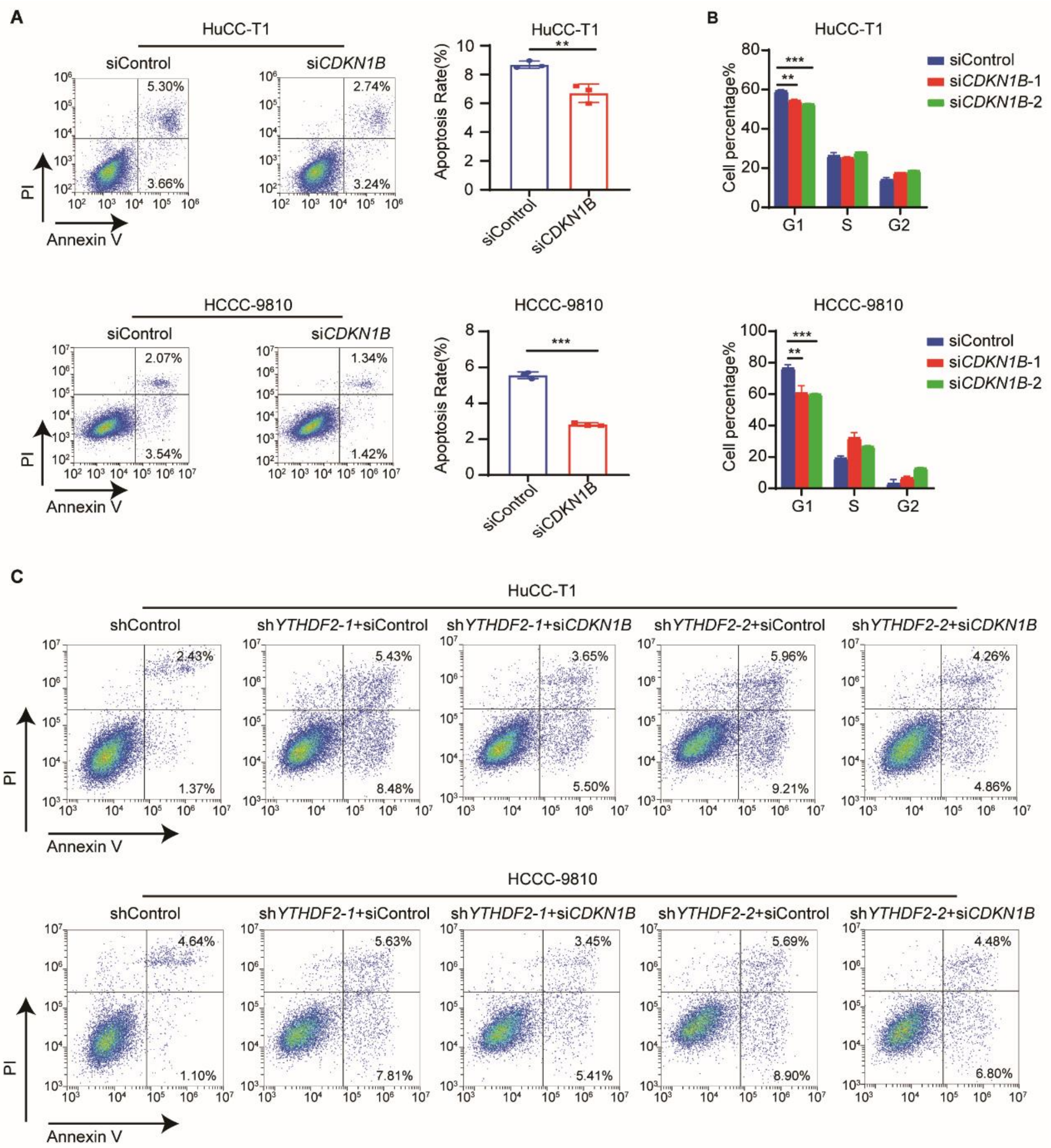

Figure S6. YTHDF2 facilitates the ICC progression by downregulating CDKN1B

A. Apoptosis analysis of HuCC-T1 and HCCC-9810 cells transfected with siControl or si*CDKN1B* for 48 hours.

B. Cell cycle analysis of HuCC-T1 and HCCC-9810 cells transfected with siControl or si*CDKN1B* for 48 hours.

C. Representative images of apoptosis analysis of HuCC-T1 and HCCC-9810 cells co-transfected with indicated shRNA or/and siRNA for 48 hours.

The data are presented as mean ± SD and compared by t-test. *$P < 0.05$; **$P < 0.01$; *** $P < 0.001$.

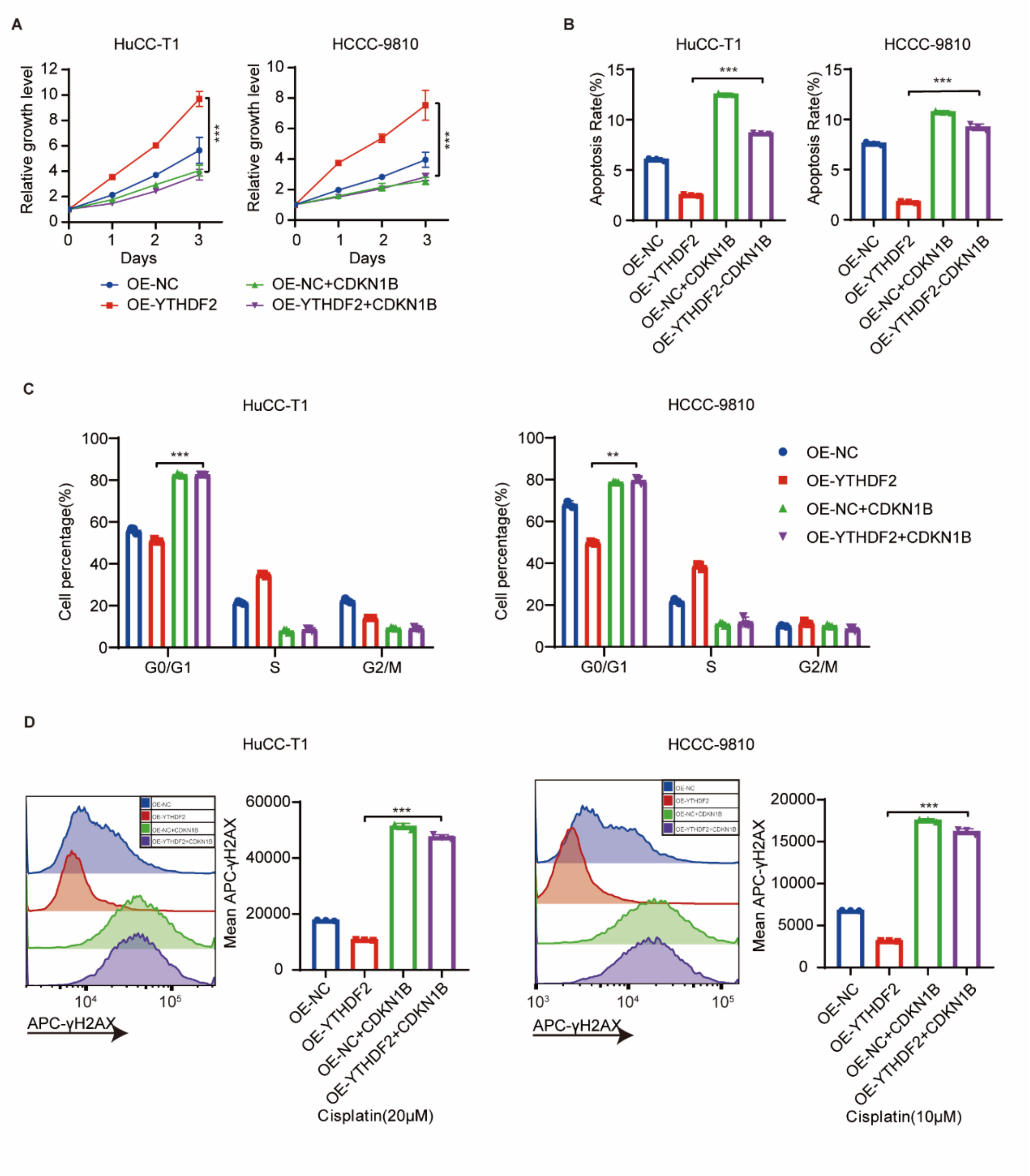

Figure S7. YTHDF2 facilitates the ICC progression and cisplatin resistance by modulating CDKN1B mRNA decay

A. Cell viability of HuCC-T1 and HCCC-9810 cells co-transfected with indicated YTHDF2 vector or/and CDKN1B vector.

B. Apoptosis analysis of HuCC-T1 and HCCC-9810 cells co-transfected with indicated YTHDF2 vector or/and CDKN1B vector.

C. Cell cycle analysis of HuCC-T1 and HCCC-9810 cells co-transfected with indicated

YTHDF2 vector or/and CDKN1B vector.

D. Representative flow cytometry analysis and mean fluorescent intensity of γ-H2AX in indicated YTHDF2 vector or/and CDKN1B vector co-transfected HuCC-T1 and HCCC-9810 cells after treatment with cisplatin (20 μM or 10 μM, respectively).

The data are presented as mean ± SD and compared by t-test. **$P < 0.01$; *** $P < 0.001$.

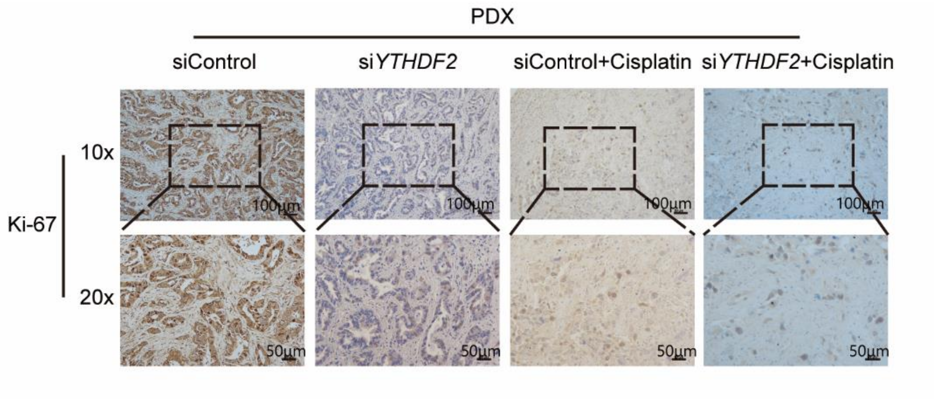

Figure S8. Representative IHC staining of Ki67 in PDX tumors with different treatments.

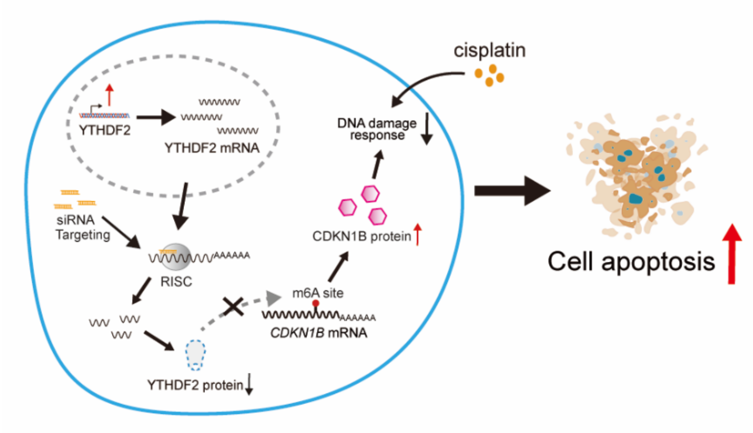

Figure S9. The graphical abstract illustrated the enhanced therapeutic efficacy of the combination treatment with *YTHDF2* siRNA and cisplatin.

Supplementary Table 1. Sequences of primers, shRNAs, and siRNAs used for experiments in this study

Names	Sequences
β-ACTIN-primer-F	CATGTACGTTGCTATCCAGGC
β-ACTIN-primer-R	CTCCTTAATGTCACGCACGAT
YTHDF2-primer-F	AGCCCCACTTCCTACCAGATG
YTHDF2-primer-R	TGAGAACTGTTATTTCCCCATGC
CDKN1B-primer-F	AACGTGCGAGTGTCTAACGG
CDKN1B-primer-R	CCCTCTAGGGGTTTGTGATTCT
CDKN1B for MeRIP-F	CAGCTTGCCCGAGTTCTACT
CDKN1B for MeRIP-R	TGTCCTCAGAGTTAGCCGGA
CDKN1B-siRNA-1-sense	GGAGCAATGCGCAGGAATA
CDKN1B -siRNA-2-sense	GCAGCAATGCGCAGGAATA
YTHDF2-siRNA-1-sense	GACCAAGAATGGCATTGCA
YTHDF2 -siRNA-2-sense	GCACAGAAGTTGCAAGCAA
YTHDF2-siRNA-3-sense	GGTAGCGGGTCCATTACTA
YTHDF2-shRNA #1	TCTGGATATAGTAGCAATTAT
YTHDF2-shRNA #2	CCACAGGCAAGGCCCAATAAT

Supplementary Table 2: Correlation between YTHDF2 expression and clinical pathological characteristics in 96 ICC patients.

Characteristics		Number of patients		*P*-value*
		Low YTHDF2 expression	High YTHDF2 expression	
Gender	Male	24	29	1.000
	Female	20	23	
Age	≤60	24	29	1.000
	＞60	20	23	
Tumor size	≤5cm	19	18	0.409
	＞5cm	25	34	
CA19-9, kU/L	≤37	24	21	0.219
	＞37	20	31	
TBIL, μmol/L	≤ 34.4	35	43	0.446
	>34.4	9	9	
TNM stage	I/II	25	18	0.040
	III/IV	19	34	
Lymphatic metastasis	Positive	6	25	0.001
	Negative	38	27	
Vascular invasion	Positive	3	14	0.014
	Negative	41	38	
Nerve invasion	Positive	3	10	0.132
	Negative	41	42	

* Chi-square test.

Supplemental Methods

Cell viability assay and colony formation assay

The transfected ICC cells were seeded in the 96-well plate at a density of 2,000 cells per well for the cell viability assay. The intensity of Luminescence was measured at 24, 48, and 72 h using the CellTiter-Glo® Luminescent Cell Viability Assay kit (Promega, Madison, WI, USA) according to the manufacturer's instructions.

For the colony formation assay, ICC cells with different treatment were seeded in the 6-well plate (1,000 cells/well), and cultured for 14 days. The cells were fixed with 4% formaldehyde and then stained with crystal violet. Cell colonies were then photographed, counted, and analyzed.

Apoptosis assay

ICC Cells with different treatment were collected and proceeded into apoptosis assay with Annexin V-FITC / PI Apoptosis Kit (#BB4101, BestBio, Shanghai, China) according to the manufacturer's instructions. After staining, the portion of apoptotic cells were analyzed via flow cytometer (BD Bioscience).

Cell cycle assay

The ICC cells were transfected with different lenti-virus or siRNA. At 48h post transfection, cells were harvested and subjected to cell cycle process using cell cycle staining Kit (#BB4104, BestBio). The data were detected by flow cytometry and analysed by ModFit LT 4.1software (Verity Software House).
